# Factors associated with occult lateral lymph node metastases in patients with clinically lymph node negative papillary thyroid carcinoma: a systematic review and meta-analysis

**DOI:** 10.3389/fendo.2024.1353923

**Published:** 2024-10-18

**Authors:** Yuan Fei, Bin Wang, Xinmin Yao, Jian Wu

**Affiliations:** Center of Breast and Thyroid Surgery, Department of General Surgery, The Third People’s Hospital of Chengdu, Affiliated Hospital of Southwest Jiaotong University, Chengdu, China

**Keywords:** papillary thyroid carcinoma, clinically lymph node negative, occult lateral lymph node metastases, factor, meta analysis

## Abstract

**Background:**

It remains unclear which category of patients with clinically lymph node negative (cN0) papillary thyroid carcinoma (PTC) might have higher risk of occult lateral lymph node metastasis (OLLNM) due to the conflicting results in previous studies. This systematic review and meta-analysis aimed to investigate factors associated with OLLNM in patients with cN0 PTC.

**Methods:**

PubMed, EMBASE, Cochrane Library and Web of Science were comprehensively searched by two independent investigators to 15 August 2022. Odds ratios (ORs) and 95% confidence intervals (CIs) were calculated for the pooled analysis. This systematic review and meta-analysis was registered in PROSPERO (CRD42022353567).

**Results:**

Fifteen eligible studies involving 8369 patients with cN0 PTC were included in this meta-analysis. We found 7 factors significantly associated with OLLNM, including male (OR, 1.47; 95% CI, 1.30 to 1.66; P < 0.001), age<45y (OR, 1.65; 95% CI, 1.31 to 2.06; P < 0.001), tumor size > 10mm (OR, 3.17; 95% CI, 2.04 to 4.93; P <0.001), tumor located in upper pole (OR, 1.81; 95% CI, 1.44 to 2.27; P <0.001), bilaterality (OR, 1.66; 95% CI, 1.37 to 2.02; P <0.001), extrathyroidal extension (ETE) (OR, 2.52; 95% CI, 1.72 to 3.68; P <0.001) and increased number of central lymph node metastasis (CLNM) (OR, 6.84; 95% CI, 5.66 to 8.27; P <0.001). The results of sensitivity analysis and subgroup analysis were similar to the pooled results. No significant publication bias was observed.

**Conclusions:**

The systematic review and meta-analysis identified 7 factors associated with OLLNM in patients with cN0 PTC. Future studies are needed to validate our results.

**Systematic review registration:**

https://www.crd.york.ac.uk/prospero, identifier CRD42022353567.

## Introduction

There were over 580000 new cases of thyroid cancer worldwide in 2020, accounting for about 3.0% of all patients with new tumor, according to World Health Organization (WHO) (1). The incidence of papillary thyroid carcinoma (PTC), the most common type of thyroid carcinoma (accounting more than 90%), is steadily increasing during last few years (2). In addition, the most frequent metastatic approach of PTC is lymph node metastasis (3). About 36.1% PTC patients are found to have cervical lymph node metastasis during primary thyroid surgery (4). Besides, the incidence of lateral lymph node metastasis (LLNM) varied from 3.1% to 65.4% based on postoperative pathological examination after neck dissection among prior studies (5).

Traditionally, PTC without obvious abnormality of cervical lymph node observed by physical examination and auxiliary examination preoperatively is defined as clinically lymph node negative (cN0) (6). It is still controversial whether prophylactic central lymph node dissection (CLND) should be performed routinely in PTC patients with cN0 (7, 8). Furthermore, the current guidelines and specialist consensus indicate that lateral lymph node dissection (LLND) should only be carried out on biopsy-proved LLNM pre- or intra-operatively, which is known as therapeutic LLND (9). However, Wada et al. observed that 39.5% papillary thyroid microcarcinoma (PTMC) patients with cN0 were found to have pathological LLNM through prophylactic LLND (10). It is reported that lateral lymph node recurrence occurs at 0.4% - 7.1% of patients who might need the second surgery if LLND was not performed at primary surgery (10, 11).

In regard to studies of risk factors related to LLNM, there was some predictive value on ultrasonic examination parameters of abnormal lymph node (12, 13). But cN0 patients can’t acquire significant benefit from preoperative ultrasound. On the other hand, early identification of occult lateral lymph node metastasis (OLLNM) in PTC patients with cN0 is of great clinical significance for optimization of surgery strategy and clinical management of patients. At present, there are significant differences in the inclusion criteria of patients, sample sizes, districts, or tumor state among these prior studies. The final results are not exactly identical. Therefore, a systematic review and meta-analysis was conducted to determine the factors associated with OLLNM in patients with cN0 PTC based on current literature search. Our investigation may provide high-quality data and evidence support for future studies that concentrate on the individualized treatment and care of patients with cN0 PTC.

## Materials and methods

This systematic review and meta-analysis was conducted according to the Preferred Reporting Items for Systematic Reviews and Meta-Analyses (PRIZMA) guideline and was reported according to Enhancing the quality and Transparency Of health Research (EQUATOR) guideline (14, 15). It has been registered on PROSPERO (CRD42022353567) (16).

### Search strategy

Two independent investigators (Yuan Fei and Bin Wang) conducted a comprehensive literature search of PubMed, EMBASE, Cochrane Library and Web of Science (from their inception dates to 15 August, 2022). There was no limitation in the publication language. The main objective of present study was to determine the factors associated in OLLNM patients.

Both Medical Subject Headings (MeSH) terms and free-text words were used to increase sensitivity for search strategy. Conference abstracts, references of associated articles and reviews were further hand-searched to identify potential eligible studies. The following search terms were used: “predict” OR “prediction” OR “factor” AND “lateral lymph node” OR “lateral neck” AND “clinical negative” OR “cN0” OR “occult” AND “papillary thyroid” OR “PTC”. Endnote X8 was used for document management.

### Eligibility criteria

The inclusion criteria of this meta-analysis were as follows: (i) studies included patients diagnosed with cN0 PTC who received CLND and LLND including at least III and IV lymph nodes at primary surgery; (ii) the outcomes, including whether lateral lymph node was metastatic, were reported.

The exclusion criteria were as follows: (i) studies included patients with family history of thyroid cancer, or history of head and neck surgery, or cervical radiation therapy. (ii) reviews, editorials, comments, and case reports; and (iii) nonhuman studies.

### Study selection

The primary outcome was defined as OLLNM diagnosed at primary thyroid surgery.

After removing duplications, all studies were selected by two researchers (Yuan Fei and Bin Wang) independently according to titles, abstracts, and full texts to evaluate potential studies strictly in terms of inclusion and exclusion criteria. Any controversy was resolved by the third researcher and by team discussion.

### Data extraction

Two researchers (Yuan Fei and Bin Wang) collected data from all eligible studies with an Excel sheet (Microsoft Corporation, Redmond, WA, USA) independently. The following data were extracted from all included studies: first author, publication year, sample size, study country, age, sex, diagnosis of tumor, surgery scope, number of OLLNM patients and pathological data investigated, including tumor size, multifocality, bilaterality, extrathyroidal extension (ETE), tumor location, and central lymph node metastasis (CLNM).

Surgery scope refers to scope of LLND, which included region II, III, IV, and V, or at least region III and IV.

### Quality assessment

The Newcastle-Ottawa quality assessment scale (NOS) was used to assess the quality of all eligible studies (17). The NOS consists of 3 parameters: selection, comparability, and outcome. Studies which earned score 6 or higher points were considered high-quality studies. Quality assessment was also completed by two independent researchers (Yuan Fei and Bin Wang).

### Statistical analysis

For clarity’s sake, percentages were rounded up or down to unit values. The statistical analysis was completed through STATA 12.0 software (StataCorp LLC, College Station, TX). We calculated odds ratios (ORs) and 95% confidence intervals (CIs) for the pooled analysis using the mantel-haenszel statistical method. The results were displayed as forest plots, and a P value <0.005 was considered statistically significant. Heterogeneity was quantified by Cochran’s chi-square test and Higgins *I²* statistic (18). A fixed-effects model was used to pool the data when P >0.05 and *I²* <50%; otherwise, a random-effects model was applied.

Sensitivity analyses were used as supplementary analyses to test the robustness of the results. The influence analysis was performed to evaluate the influence of each study on the combined ORs by removing one study at a time. Besides, the random-effect model was used to further validate the results which were primarily calculated through the fixed-effect model. The subgroup analyses were also performed to explore whether country and scope of LLND in included original studies would affect the pooled results. Possible publication bias was evaluated via the Begg’s and Egger’s funnel plot when the number of included original studies were above 10.

## Results

### Search process and study characteristics

**Figure 1:**
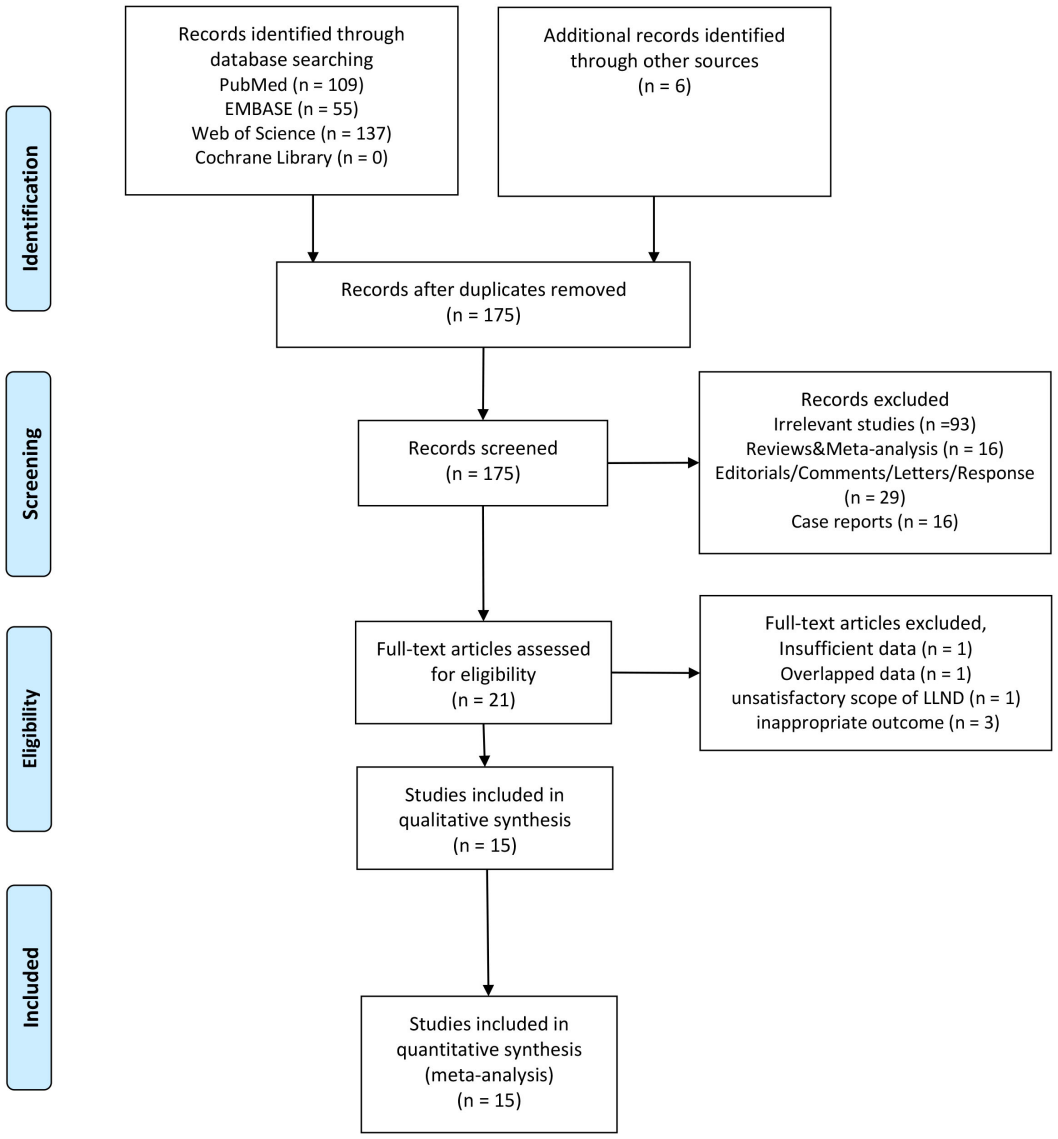
Study flow diagram.

As shown in **Figure 1**, a total of 307 potential studies were identified from database searches. After removing duplicates, 175 studies were left to be screened for titles and abstracts. Then, 154 studies were excluded according to inclusion and exclusion criteria. After that, 21 remaining studies were selected for assessing eligibility and eventually 15 studies with a total of 8369 patients were enrolled in the meta-analysis (10, 19–32).

They were all observational studies with the sample size varying from 62 to 3219 patients. The pooled incidence of OLLNM was 41.0% (3434/8369). As for quality assessment, all studies earned at least 6 points. The study characteristics and quality assessment scores are summarized in **Table 1**.

**Table 1:** Characteristics of included studies.

Author	Year	country	period	No. patients	Age (years) Mean±SD (range)	Male, (%)	Diagnosis	Surgery scope	No. OLLNM	Risk factors	NOS score
Zhou	2022	China	01/2016-12/2017	388	43.5 (13.6)	116 (29.9)	cN0 PTC	TT+ CLND + LLND	181	gender; tumor location; multifocality; ETE; HD; CLNM	8
Wang	2021	China	01/2013- 12/2018	1033	N.R.	287 (27.8)	cN0 PTC	TT+ CLND + LLND	485	gender; HD; ETE; Bilaterality	7
Chen	2021	China	01/2015-12/2019	1107	15-80	279 (25.2)	cN0 PTC	TT/LT+ CLND (+ LLND)	117	gender; tumor size; multifocality; bilaterality; ETE; HD; CLNM	7
Hu	2018	China	01/2013- 12/2016	783	42.4 (13.1)	207 (26.4)	cN0 PTC	TT+ CLND + LLND	371	age; gender; tumor size; tumor location; ETE; multifocality; Bilaterality; HD; CLNM	7
Tao	2017	China	01/2008-06/2015	66	43.5 (19-69)	13 (19.7)	cN0 unilateral PTC	TT/LT+ CLND + LLND	27	age; gender; tumor size; ETE; multifocality	8
Goran	2017	Serbia	2004-2013	111	49.42 (12.53)	17 (15.3)	cN0 PTMC	TT+ CLND (+ LLND)	8	multifocality; Bilaterality	8
An	2017	China	01/2006- 12/2013	138	41 (18-71)	33 (23.9)	cN0 PTC	TT/LT+ CLND+ III+IV + (II+V)	67	age; gender; tumor location; tumor size; ETE; multifocality; CLNM	7
Noda	2015	Japan	2001-2011	246	54.6 (15.3)	36 (14.6)	cN0 PTC	TT/sLT/LT+ CLND+ LLND	85	age	7
Patron	2013	France	01/1974- 12/2006	173	39.9 (14.3)	38 (22.0)	cN0 PTC (> 10mm)	TT+ CLND+LLND	34	age; gender; tumor size; multifocality; Bilaterality; ETE	8
Ito	2013	Japan	1987-2005	3219	49.5 (11-87)	288 (9.0)	cN0 PTC	TT/LT+CLND+LLND	1846	gender; multifocality; tumor size; ETE	8
Ducoudray	2013	France	04/2003-04/2011	603	49 (17-85)	109 (18.1)	cN0 PTC	TT/cTT + CLND+LLND	51	gender; tumor size; multifocality; Bilaterality; tumor location; ETE; CLNM	8
Lim	2011	Korea	04/2008-05/2009	62	47.1	13 (21.0)	cN0 PTC	TT+ CLND+LLND	34	Gender; multifocality; ETE	8
Vergez	2010	France	2000-2006	90	45 (16)	19 (21.1)	cN0PTC (>10mm)	TT+ CLND+LLND	28	Gender; multifocality	6
Bonnet	2008	France	01/2000-12/2005	115	44.5 (18-73)	16 (13.9)	cN0PTC (<20mm)	TT+ CLND+ III+IV+ (II+V)	27	tumor size	7
Wada	2003	Japan	1988-1998	235	48 (17-72)	25 (10.6)	cN0 PTMC	TT/sLT/LT+ CLND (+ LLND)	73	Tumor location; multifocality; Bilaterality	7

No., number; cN0, clinically lymph node negative; PTC, papillary thyroid carcinoma; PTMC, papillary thyroid microcarcinoma; TT, total thyroidectomy; LT, lobectomy; sLT, subtotal thyroidectomy; cTT, completion thyroidectomy; CLND, central lymph node dissection; LLND, lateral lymph node dissection; OLLNM, occult lateral lymph node metastasis; HD, Hashimoto’s disease; ETE, Extrathyroidal extension; CLNM, central lymph node metastasis; N.R., not reported; NOS, Newcastle-Ottawa quality assessment scale.

### Meta-analysis

**Figure 2:**
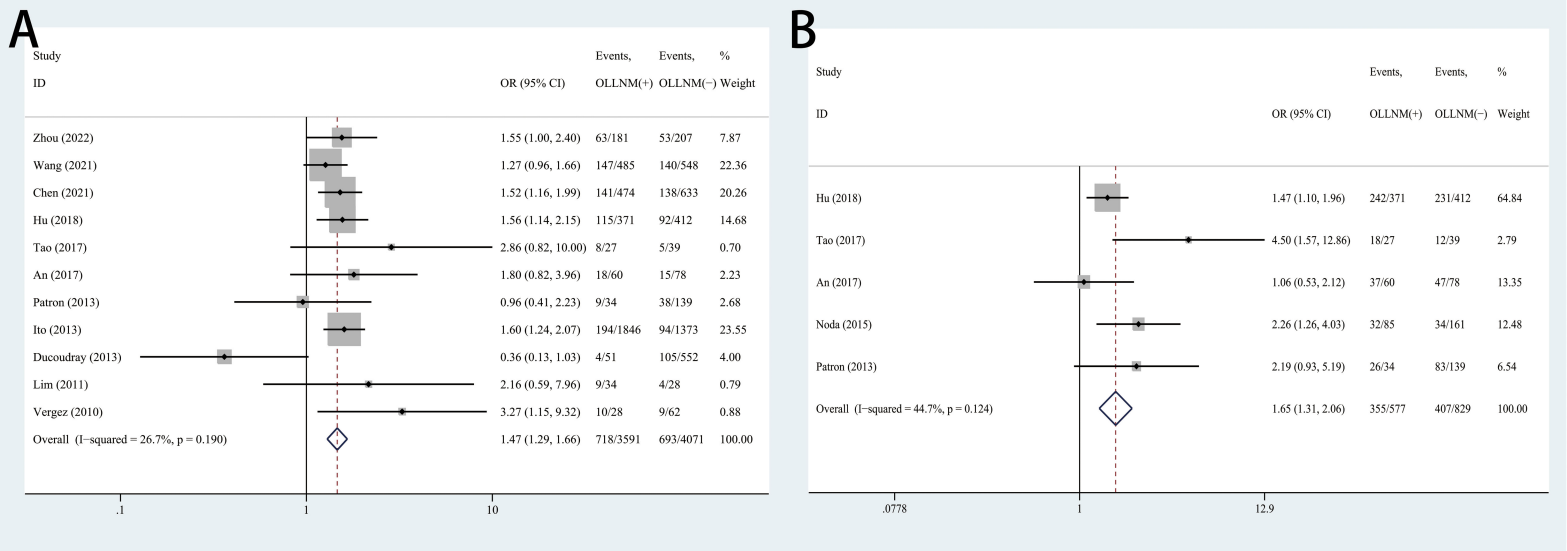
Forest plots of associations between gender male **(A)**, age under 45 years **(B)** and occult lateral neck lymph node metastasis (OLLNM) in clinically lymph node negative (cN0) papillary thyroid carcinoma (PTC).

A total of 11 studies reported the impacts of gender on outcomes. The fixed-effects model was applied due to low heterogeneity (*I^2^ =* 26.7%, P=0.190), and the pooled results indicated that the risk of OLLNM was significant higher in male patients (OR, 1.47; 95% CI, 1.29 to 1.66; P < 0.001) (**Figure 2A**). Fixed-effects model was also adopted to investigate the impacts of age on OLLNM (*I^2^ =* 44.7%, P =0.124). Pooled analysis of 5 studies showed that the risk of OLLNM was significantly higher in patients younger than 45 years (OR, 1.65; 95% CI, 1.31 to 2.06; P < 0.001) (**Figure 2B**).

**Figure 3:**
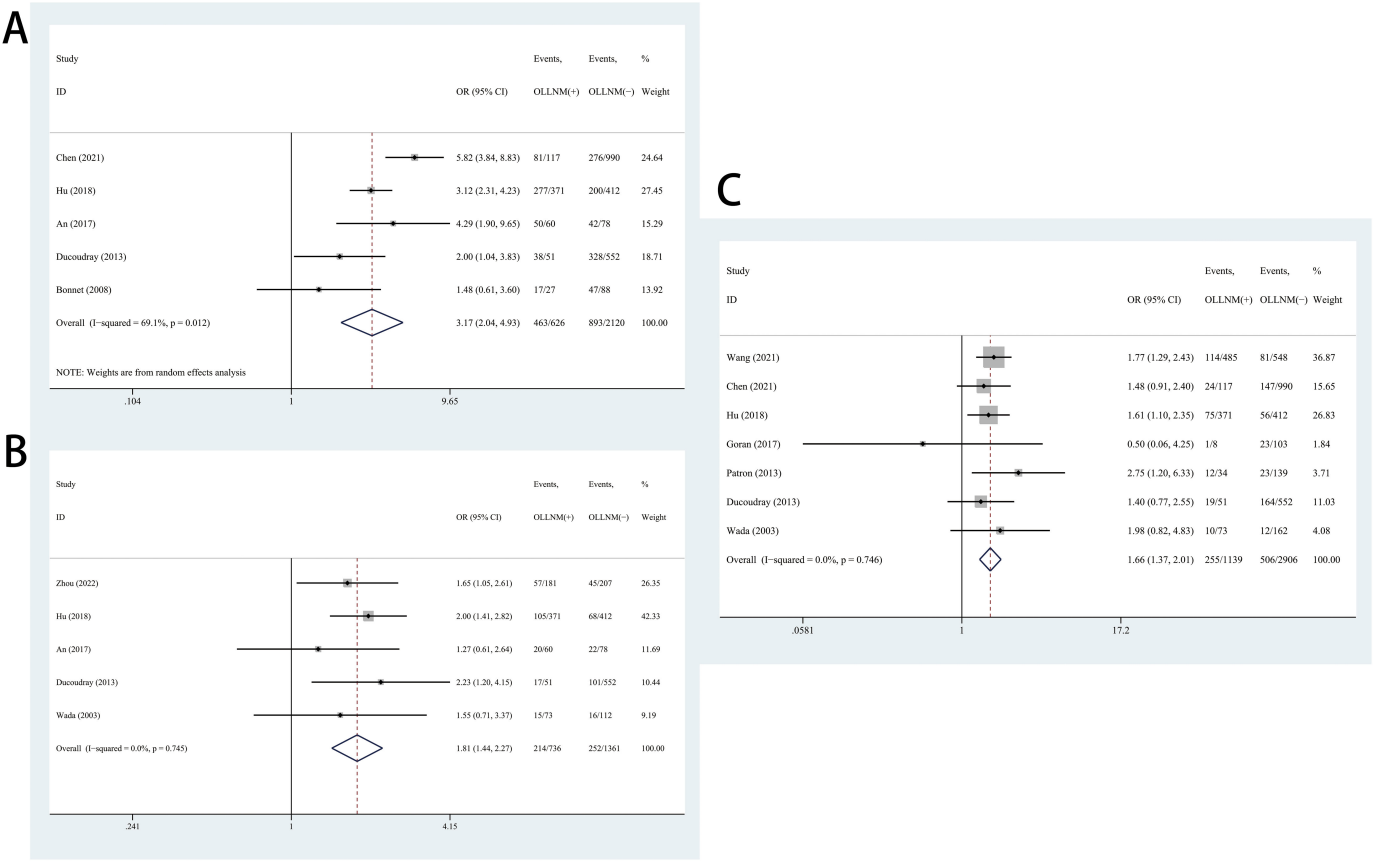
Forest plots of associations between tumor size over 10mm **(A)**, tumor located in upper pole **(B)**, bilateral PTC **(C)** and occult lateral neck lymph node metastasis (OLLNM) in clinically lymph node negative (cN0) papillary thyroid carcinoma (PTC).

The pooled results of 5 studies suggested significant higher risk of OLLNM when tumor size over 10mm compared to PTMC (OR, 3.17; 95% CI, 2.04 to 4.93; P <0.001) with random effects model due to high heterogeneity (*I^2^ =* 69.1%, P =0.012) (**Figure 3A**). In addition, patients with tumor located in upper pole have higher risk of OLLNM than those with tumor located in other poles (OR, 1.81; 95% CI, 1.44 to 2.27; P <0.001; *I^2^ =* 0.0%, P=0.745) (**Figure 3B**). A total of 7 studies reported the influence of bilateral PTC on the risk of OLLNM. Pooled analysis demonstrated that the risk of OLLNM was significantly higher when tumor was distributed in bilateral lobes of the thyroid than distributed in unilateral lobe (OR, 1.66; 95% CI, 1.37 to 2.01; P <0.001; *I^2^ =* 0.0%, P =0.746) (**Figure 3C**).

**Figure 4:**
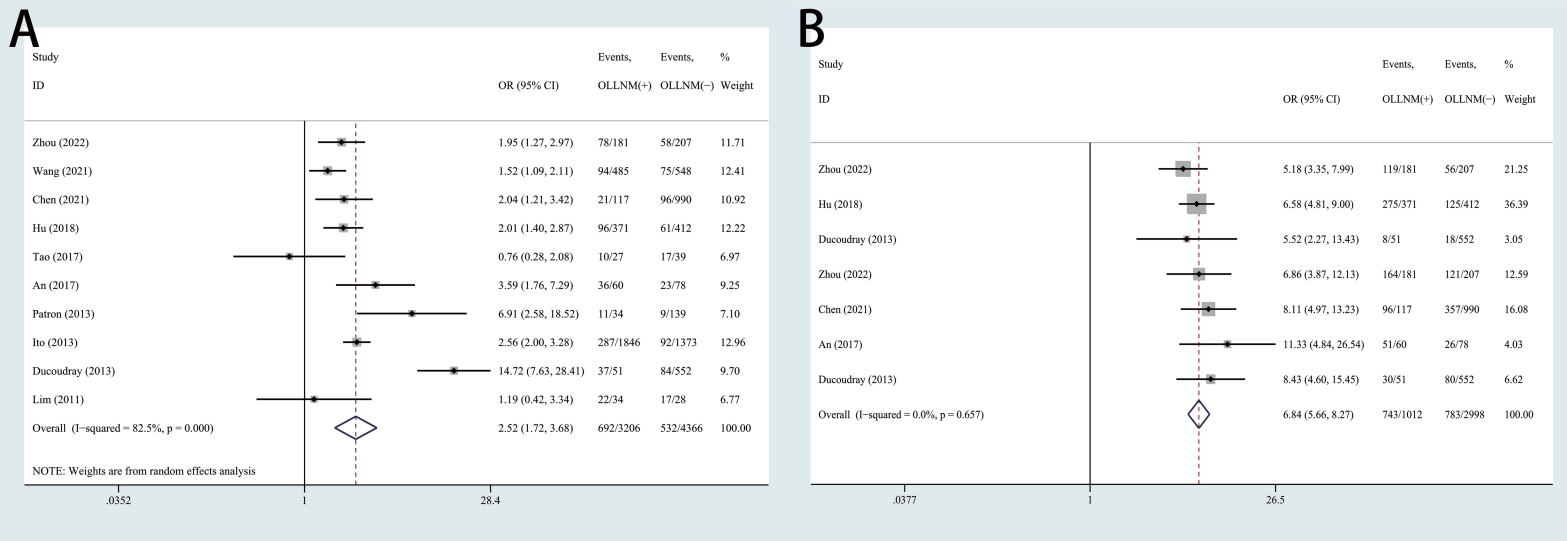
Forest plots of associations between extrathyroidal extension (ETE) **(A)**, increased number of central lymph node metastasis (CLNM) **(B)** and occult lateral neck lymph node metastasis (OLLNM) in clinically lymph nod negative (cN0) papillary thyroid carcinoma (PTC).

In the pooled analysis of 10 studies, the risk of OLLNM was significantly higher in cN0 PTC patients with ETE than those without ETE (OR, 2.52; 95% CI, 1.72 to 3.68; P <0.001) with a random effects model (*I^2^ =* 82.5%, P<0.001) (**Figure 4A**). In addition, 7 studies reported the impacts of CLNM on the OLLNM. A fixed-effects model was used considering the low heterogeneity (*I^2^ =* 0.0%, P=0.657). The risk of OLLNM was significant higher in patients with increased number of CLNM (OR, 6.84; 95% CI, 5.66 to 8.27; P <0.001) (**Figure 4B**).

### Sensitivity and subgroup analyses

The results of influence analysis showed that the pooled ORs were robust. (**Supplementary Material 1**, **Supplementary Figures S1**-**S7**). As for the results generated through fixed-effect model, the additional random-effect model analyses also verified the stability of these results (male: OR, 1.48; 95% CI, 1.25 to 1.75; age<45y: OR, 1.80; 95% CI, 1.24 to 2.60; tumor located in upper pole: OR, 1.81; 95% CI, 1.44 to 2.27; bilaterality: OR, 1.67; 95% CI, 1.38 to 2.03; CLNM: OR, 6.80; 95% CI, 5.63 to 8.21);. The pooled outcomes were stable and reliable for these factors (**Supplementary Material 1**, **Supplementary Figures S8**-**S12**).

High heterogeneities were observed among the studies that reported the impacts of tumor size and ETE on OLLNM. Therefore, subgroup analyses were performed to explore the effects of diverse regions and surgical scopes of lateral lymph node dissection (LLND) on the pooled outcomes. However, the subgroup outcome of studies in which III-IV LLND showed no significant association between OLLNM and tumor size with less heterogeneity (OR, 1.40; 95% CI, 0.92 to2.13; P=0.118; *I^2^ =* 0.0%, P=0.545). No significant source of heterogeneity was found in regards to ETE. Detailed results were presented in **Table 2**.

**Table 2:** Subgroup analysis of association between two clinicopathological predictors and OLLNM in cN0 PTC.

	No. of study	OR (95% CI)	Log-rank *P* value	*I^2^* (%)	*P* value
Tumor size					
Country					
China	3	1.83 (1.30,2.57)	0.046	67.6%	0.001
Non-China	2	1.23 (0.85,1.79)	0.884	0.0%	0.274
LLND scope					
II-V	3	1.71 (1.18,2.49)	0.017	75.5%	0.005
III-IV	2	1.40 (0.92,2.13)	0.545	0.0%	0.118
ETE					
Country					
China	6	1.59 (1.33,1.90)	0.622	0.0%	<0.001
Non-China	4	2.78 (1.55,4.96)	0.004	77.8%	0.001

No., number; LLND, lateral lymph node dissection; ETE, Extrathyroidal extension.

### Publication bias

The funnel plots were symmetric (**Supplementary Material 1**, **Supplementary Figures S13**, **S14**) and the Begg’s and Egger’s test did not show any evidence of publication bias in the meta-analysis of association of OLLNM with sex (Begg’s test: *P*=0.436; Egger’s test: *P*=0.951), ETE (Begg’s test: *P*=0.858; Egger’s test: *P*=0.609).

## Discussion

The current research on thyroid malignancy mainly includes surgical instruments, surgical resection scope, protection of parathyroid function and tumorigenic pathways, etc (33–35). Zhan et al. has reported predictors of OLLNM in N0/N1a PTC patients (36). In the present meta-analysis, cN0 PTC patients were singled out for study. On the basis of statistical analysis, incidence rate of OLLNM was 41.0%, which was consistent with results of previous studies (7.2%-57.3%) (24, 28). The factors significantly associated with OLLNM consisted of male, age< 45y, larger tumor size, PTC located in upper lobe, bilaterality, ETE and CLNM. The results of sensitivity analyses, subgroup analyses and publication bias suggested the stability and robustness of our results.

Cervical lymph node metastasis has been reported to be an important related factor of local recurrence of PTC patients. The study from Bardet et al. suggested that 6.4% (35/545) of PTC patients developed lymph node recurrence after first operation (37). Lee et al. further argued that the N stage was inversely associated with recurrence-free rate. In particular, patients with pathological N1b PTC were at 2.8 times risk of recurrence compared to those with N1a (38). The thorough dissection at primary surgery has a direct and important impact on the prognosis of patients with cN0 PTC. As a result, it is necessary to identify factors associated with OLLNM in cN0 PTC patients, which could contribute to overall therapy and management of patients.

Ding et al. reported that there was a high rate of LLNM in male patients based on a retrospective cohort study (39). In addition, Zhuo et al. carried out a multivariable regression analysis and suggested that male patients may experience 4.8 times more than female in LLNM (40). Cao et al. investigated molecular mechanisms of underlying LNM by proteomics approach, and found that the elevated levels of extracellular matrix (ECM)-associated proteins might be related to more odds of LNM in male patients with PTMC, which is partly responsible for the results of above studies (41). Based on the meta-analysis, we also concluded that male was a significant factor associated with OLLNM in cN0 PTC patients.

Ito et al. performed a retrospective study with 1235 low-risk PTMC patients and reached a conclusion that young age was significant associated with novel appearance of LNM (<40 years vs. 40-59 years, P=0.0005) (42). Another study that enrolled patients with various T stage demonstrated that there was a reverse association between age at diagnosis and LNM rates with each T stage (43). Therefore, patients younger than 45 years might be monitored for thyroglobulin and cervical lymph node ultrasound more frequently after surgeries than others to detect LLNM as early as possible.

Tumor size over 10mm, presence of ETE, bilateral PTC and PTC located in upper lobe were all significant factors in previous studies which determined LLNM or OLLNM as primary outcome (4, 5, 36). The results of present meta-analysis further demonstrated that these factors were valuable in predicting OLLNM of cN0 patients. Considering that the personalized therapy provides an important basis for the improvement of prognosis, those patients with these factors should be paid more attention to in clinical practice.

The origins of heterogeneity were not identified in subgroup analysis. It was worth noting that tumor size over 10mm was significantly associated with OLLNM only in studies including patients received II-V LLND. The unstable result may come from limited included researches, in addition lateral lymph node metastasis cannot be estimated accurately via III-IV LLND. Therefore, the role of lymph node dissection scope in association between tumor size and OLLNM need further investigation. In addition, ETE was significantly associated with OLLNM in both Chinese and non-Chinese patients. The source of heterogeneity was from non-China studies. These articles were published earlier, therefore definition, diagnosis and treatment maybe not consistent with later researches, which led to obvious increased heterogeneity. Because various countries may have differences in disease spectrum, age of onset and other baseline characteristics and treatment methods for cN0 PTC patients. Therefore, the subgroup analysis was performed to explore the stability of the pooled results, the possible sources of heterogeneity, and the potential influence of national and regional factors on the results.

According to our analysis, the risk of OLLNM was 6.84-fold higher in cN0 PTC patients with increased number of CLNM. Kim et al. reported that >2mm CLNM has a significant impact on higher rates of recurrence of lateral neck nodes metastasis in PTC patients (44). Therefore, clinicians should pay more attention to patients with CLNM. Prophylactic CLND could be considered at first surgery for PTC patients. Future studies with large sample size are warranted to determine the association between size of metastasis foci in CLN and risk of OLLNM in patients with cN0 PTC.

Ito et al. put forward that clinically apparently rather than pathologically confirmed LLNM was an independent predictor of worse disease-free survival of PTC (45). OLLNM was not related to clinical impact significantly, hence, prophylactic LLND was not recommend in most international guidelines. However, patients with OLLNM may face additional lateral lymph node surgery in follow-up. In addition, 2015 ATA guideline recommended that cervical ultrasound should be performed to evaluate thyroid bed, cervical and lateral nodal compartment at 6-12 months (9). Therefore, more frequent follow-up rather than immediate prophylactic LLND should be considered for patients with more factors associated with OLLNM. Radioiodine therapy could be also considered to decrease risks of LLN recurrence. However, to avoid the influences of radioiodine therapy on OLLNM and to reduce the heterogeneity of final results, the studies investigating factors of LLN recurrence after first thyroid and CLND operation were not included in present meta-analysis.

Several limitations of this meta-analysis should be acknowledged. First, high heterogeneities were found in some pooled results, which may be attributed to retrospective study designs and diverse types of populations, definitions of cN0, and surgical scopes. In addition, meta regression analysis was unsuited to perform because of limited studies. Second, not all enrolled patients underwent II-V lateral lymph node dissection, which may underestimate the incidence of OLLNM and lead to bias of results. Third, it was reported that incidence of skip lateral lymph node metastasis was7.4%-9.7% (46, 47). However, skip metastasis can’t be divided from OLLNM in this meta-analysis. Similarly, the underestimation of incidence of OLLNM could affect final results. Besides, studies identifying significant factors associated with OLLNM are probably more prone to be published than those which did not identify significant related factors, which might induce a potential bias in the pooled estimates. Finally, all included studies were published in English, which indicated that some eligible studies might have been excluded because of different languages.

## Conclusion

In conclusion, we identified 7 clinicopathologic features which were significantly associated with the increased risks of OLLNM: male, age<45y, tumor size > 10mm, tumor located in upper pole, bilaterality, ETE and increased number of CLNM. The status of lateral lymph node in those patients with more factors should be paid more attention to during follow-ups. Our conclusions need to be verified in the future studies.

## Data Availability

The original contributions presented in the study are included in the article/**Supplementary Material**. Further inquiries can be directed to the corresponding author.
