# Supplementary material for: Factors associated with occult lateral lymph node metastases in patients with clinically lymph node negative papillary thyroid carcinoma: a systematic review and meta-analysis
Source: Front Endocrinol (Lausanne). 2024 Oct 18;15:1353923. doi: 10.3389/fendo.2024.1353923 (PMC11527613; doi:10.3389/fendo.2024.1353923)
Supplement: Supplementary file 1 [file DataSheet1.docx]

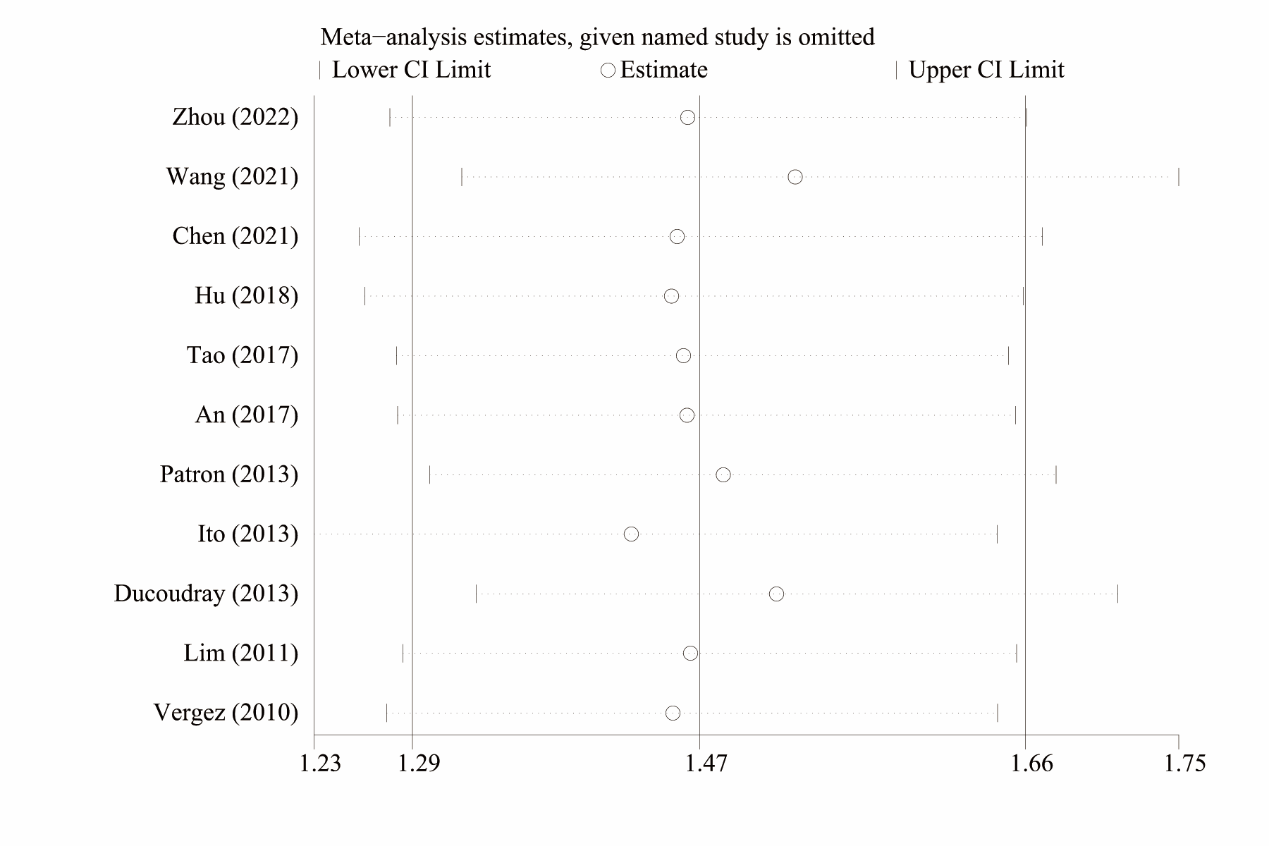


Figure S1. Sensitivity analysis for the association of male with risk of OLLNM.


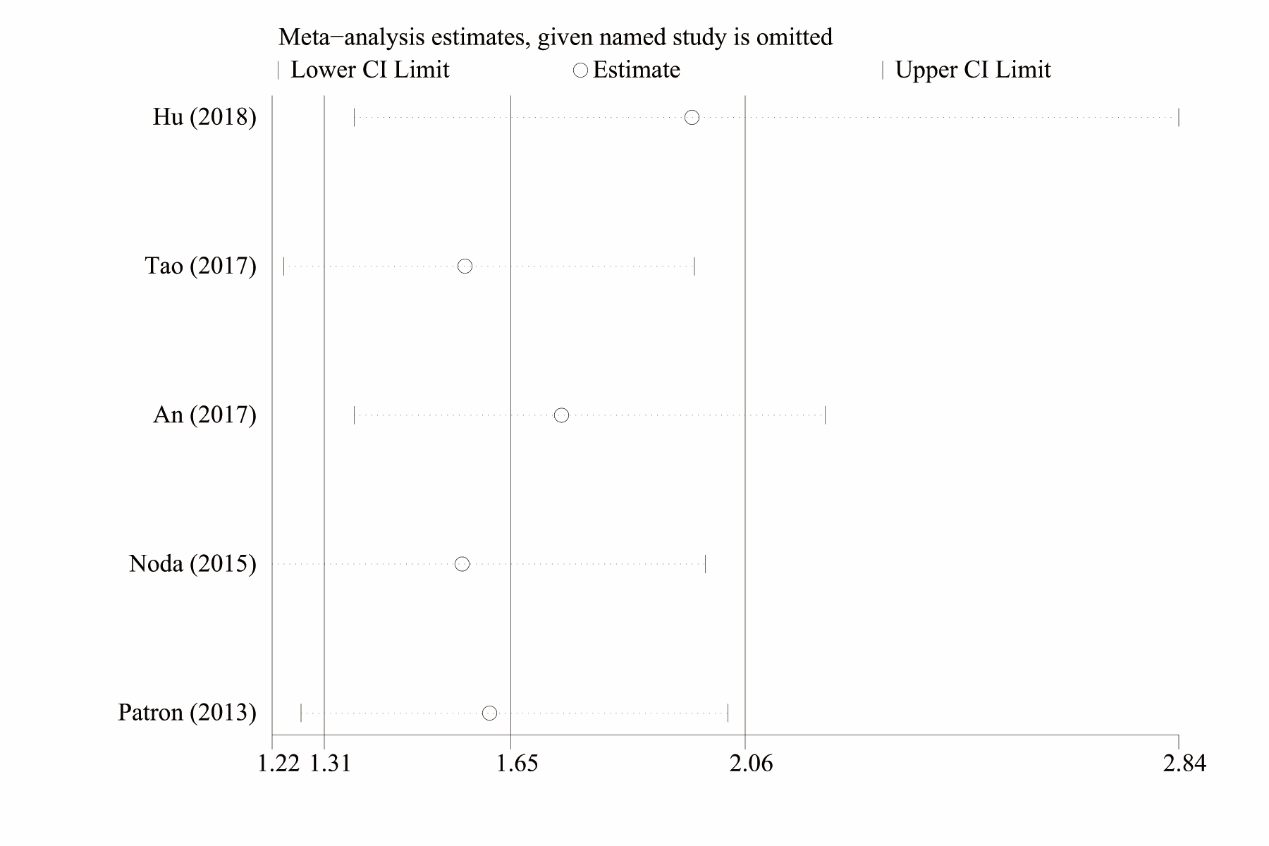


Figure S2. Sensitivity analysis for the association of age under 45 years with risk of OLLNM.


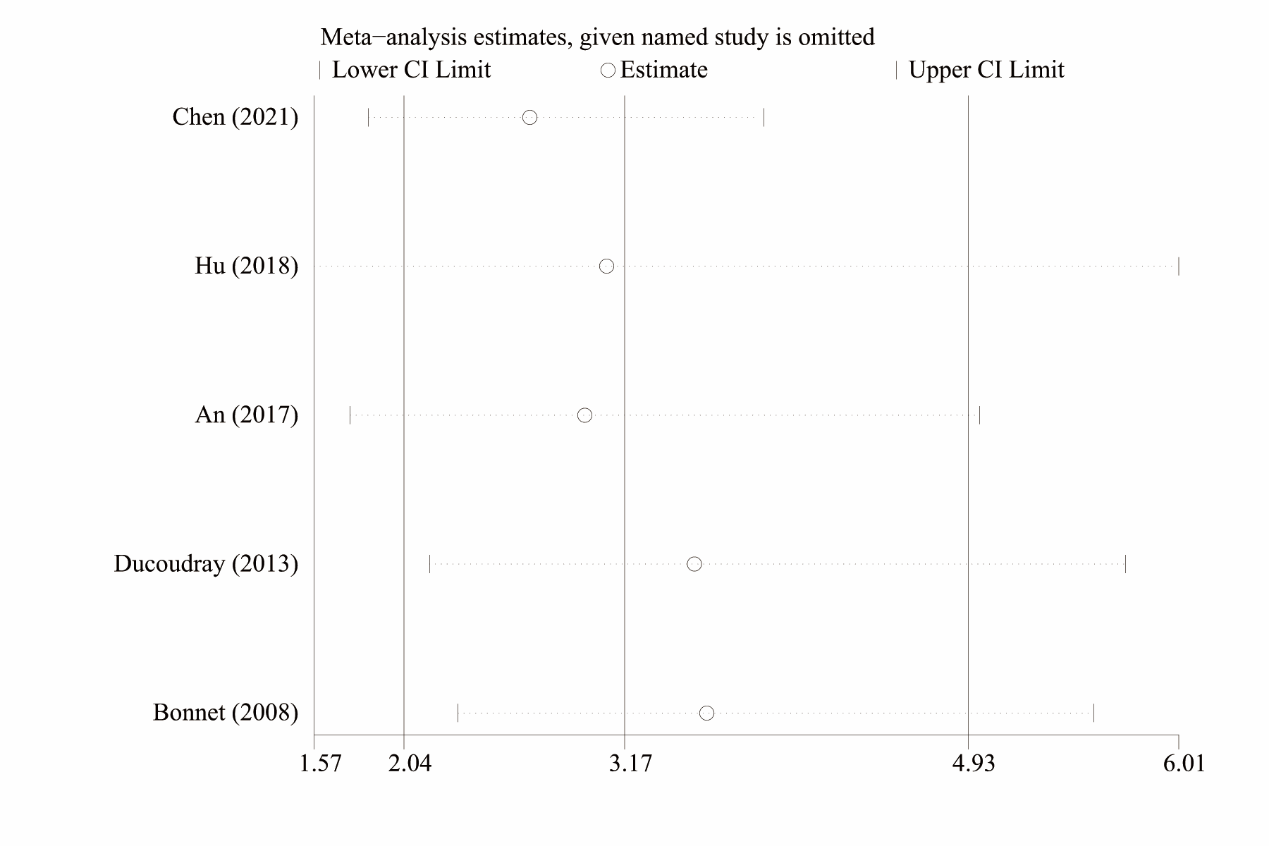


Figure S3. Sensitivity analysis for the association of tumor size over 10mm with risk of OLLNM.


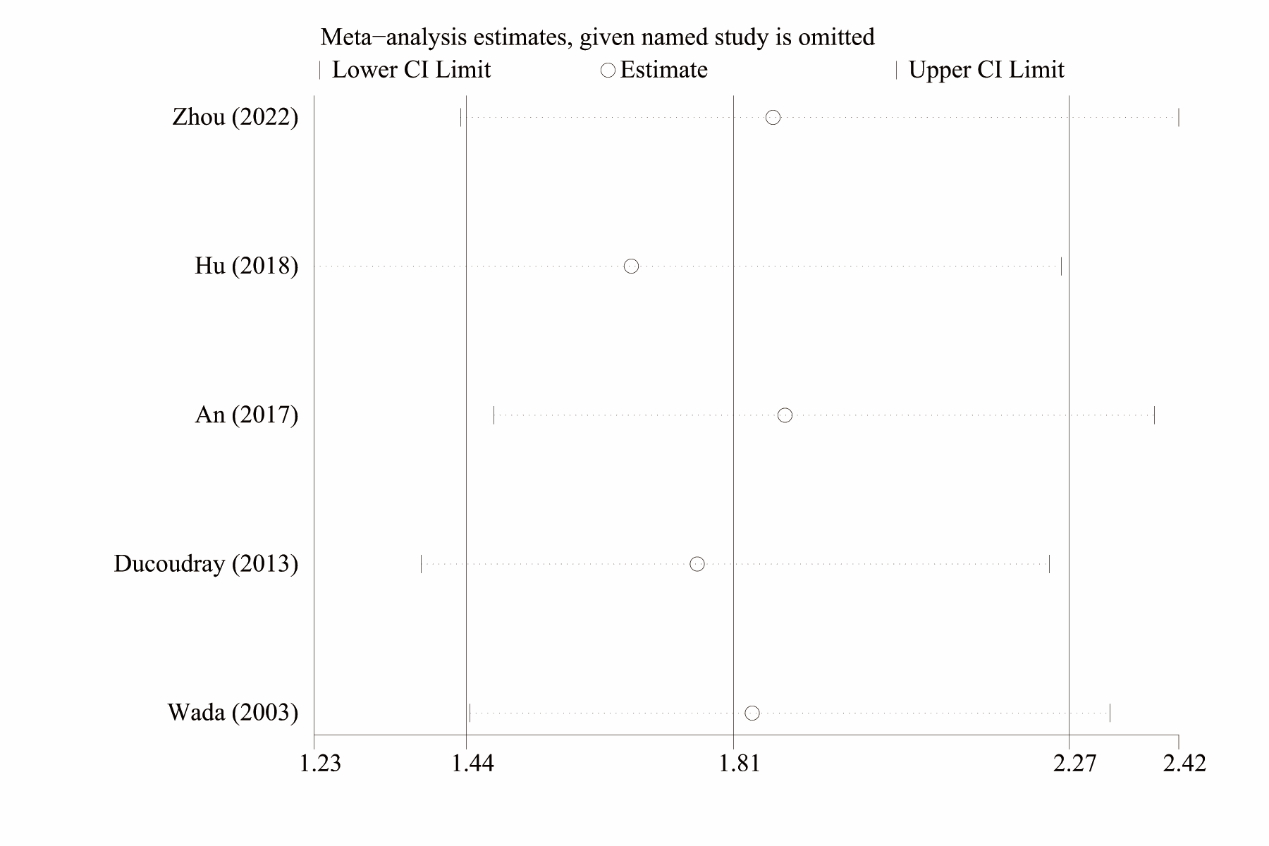


Figure S4. Sensitivity analysis for the association of tumor located in upper pole with risk of OLLNM.


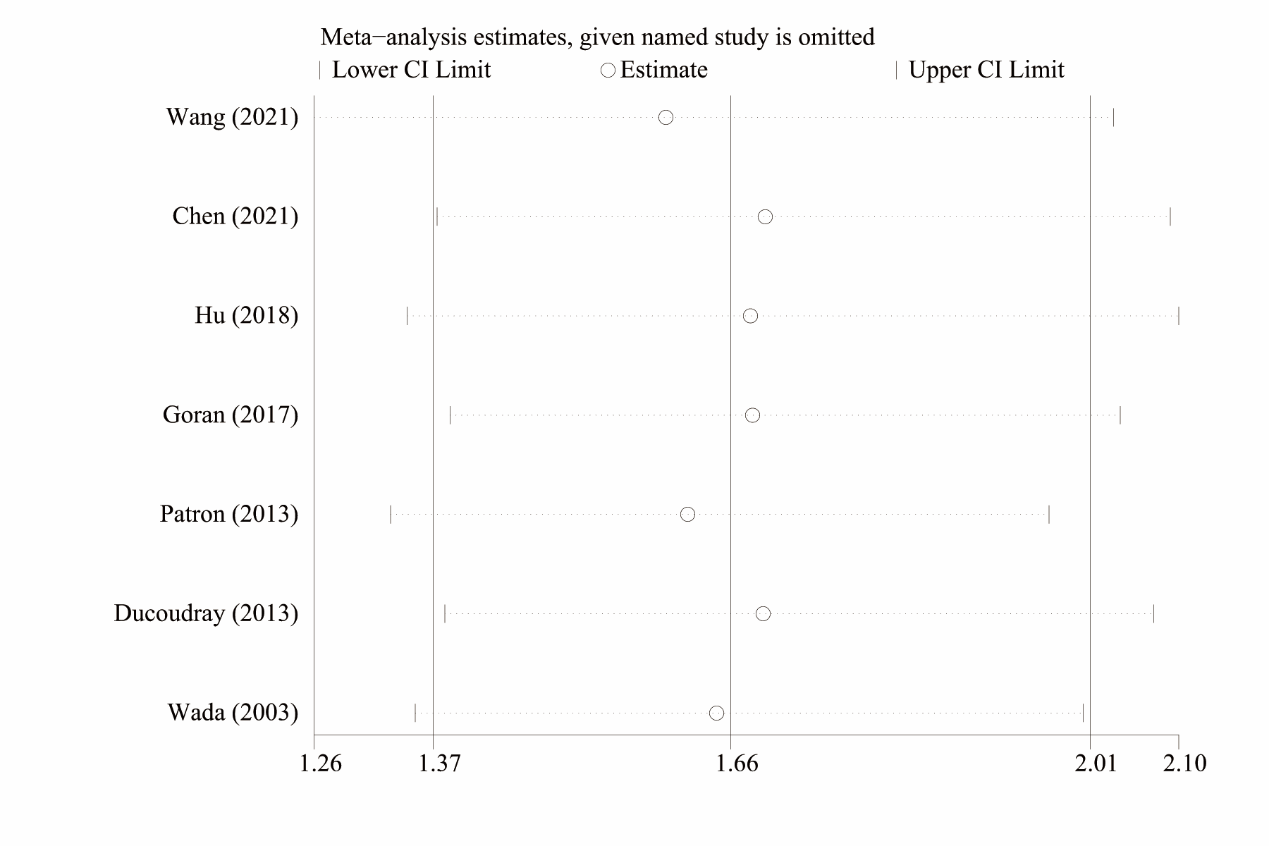


Figure S5. Sensitivity analysis for the association of bilateral PTC with risk of OLLNM.


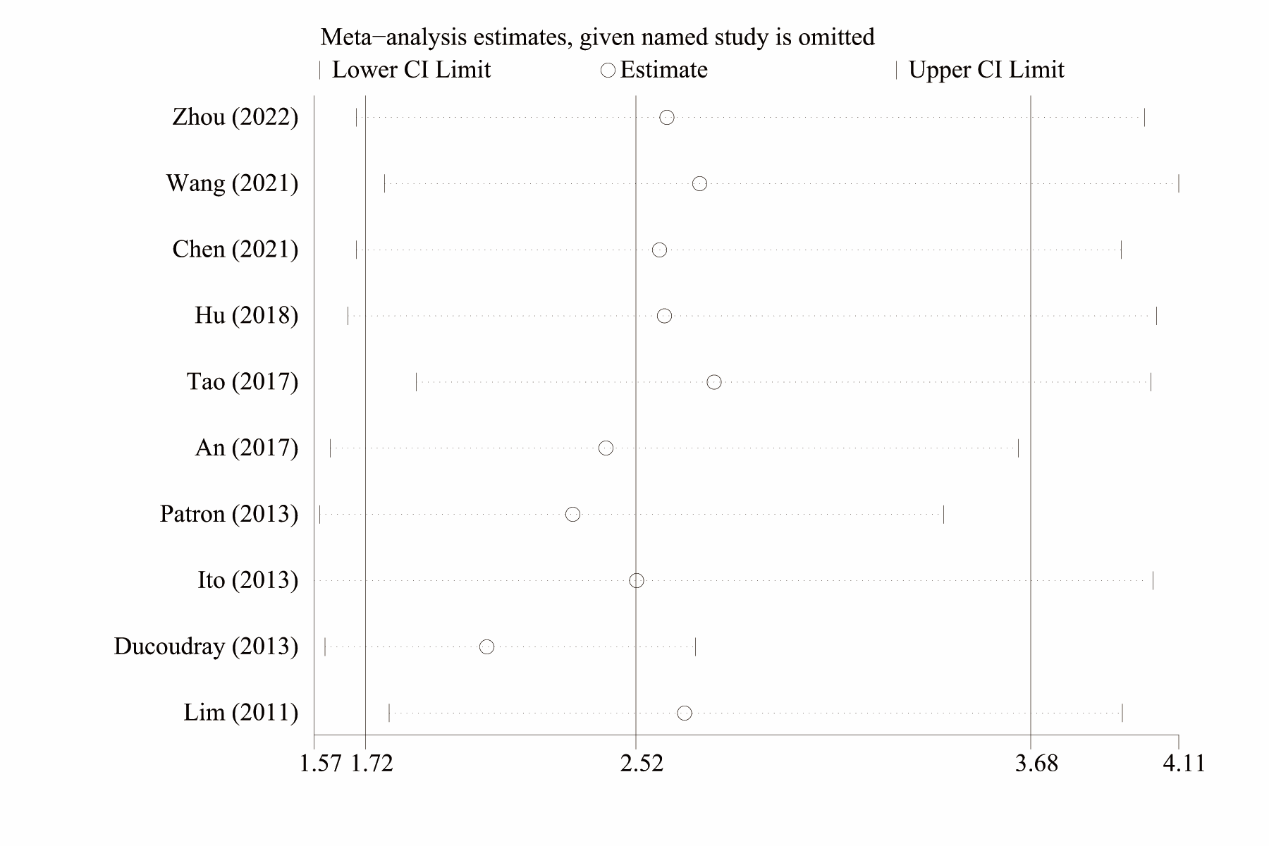


Figure S6. Sensitivity analysis for the association of extrathyroidal extension (ETE) with risk of OLLNM.


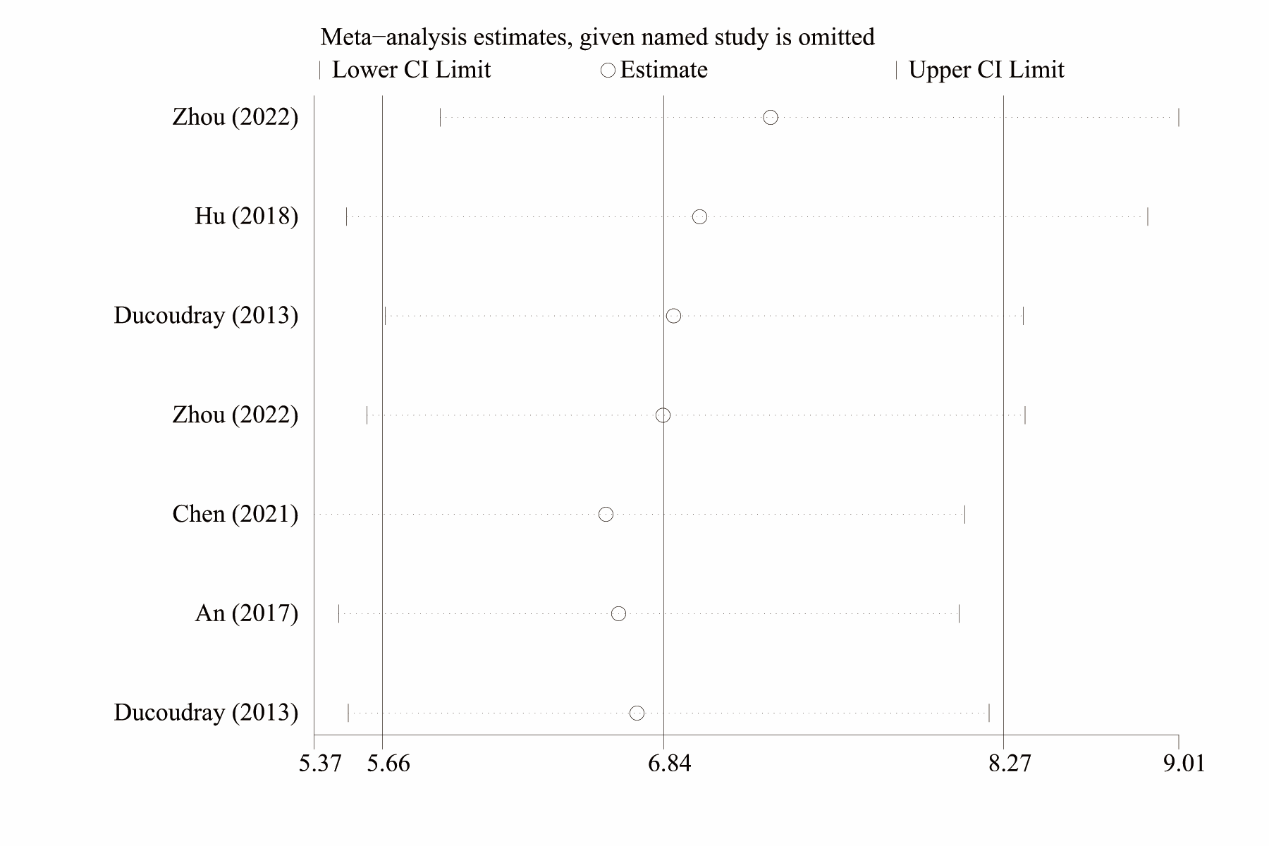


Figure S7. Sensitivity analysis for the association of increased number of central lymph node metastasis (CLNM) with risk of OLLNM.


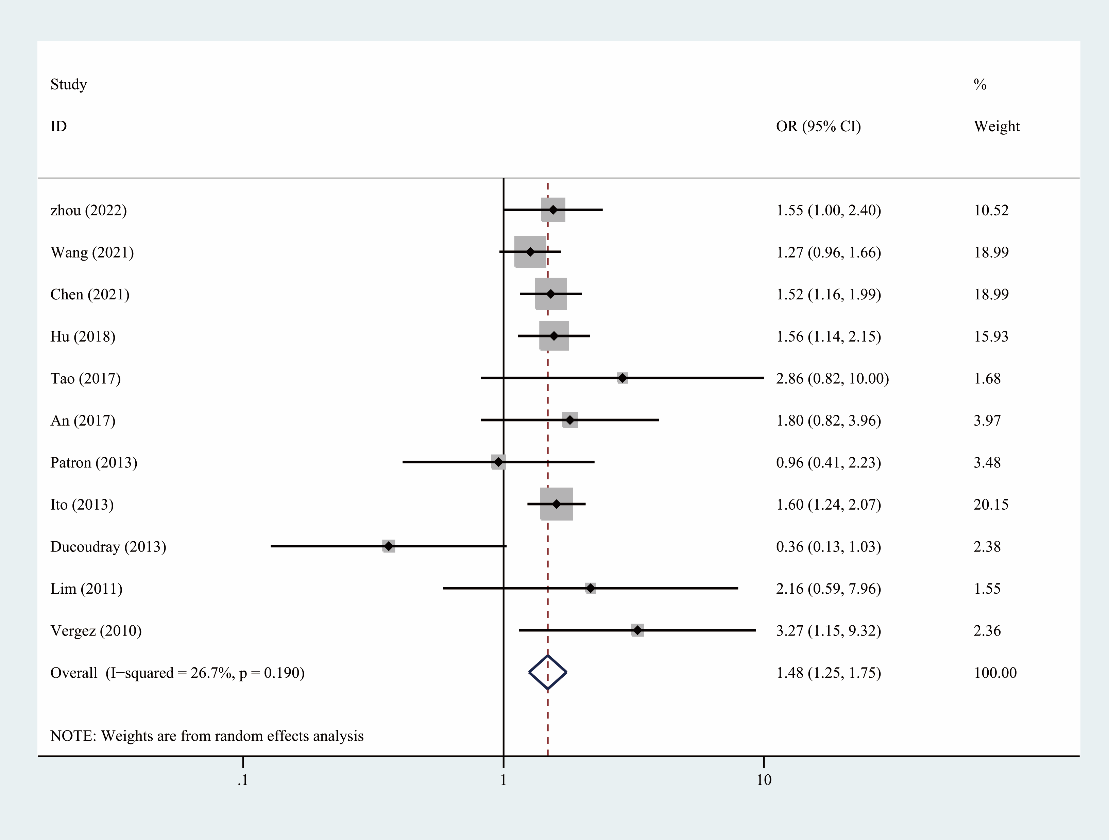


Figure S8. A random-effect model for the association of male with risk of OLLNM.


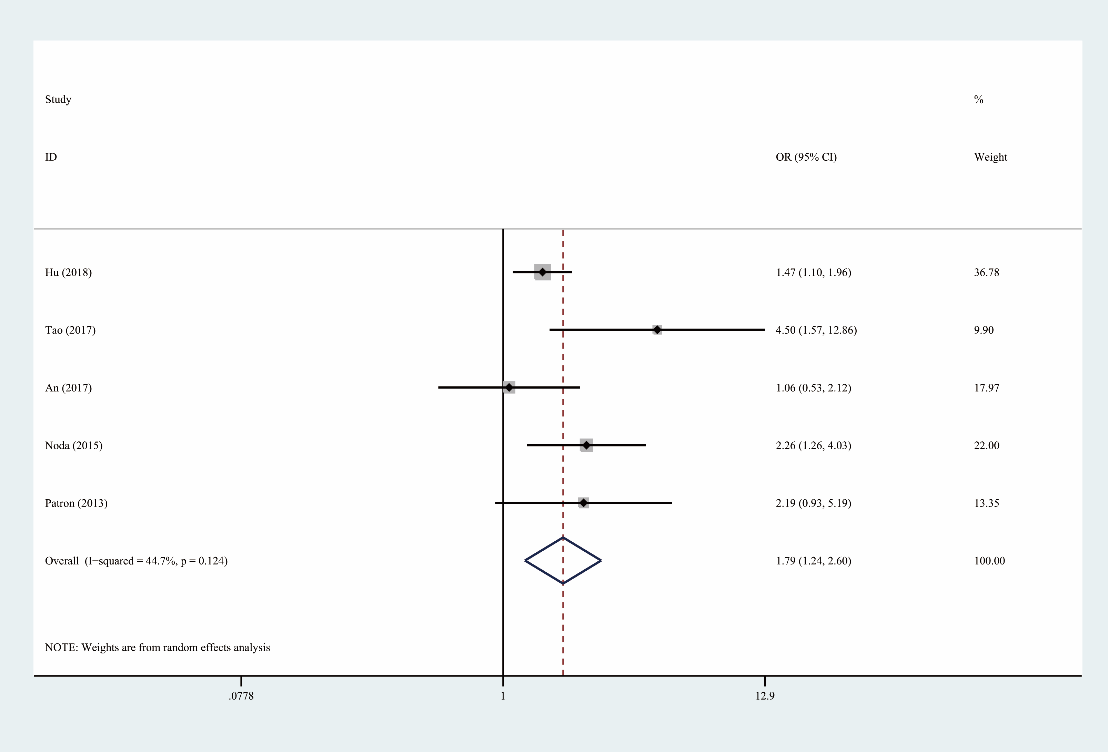


Figure S9. A random-effect model for the association of age under 45 years with risk of OLLNM.


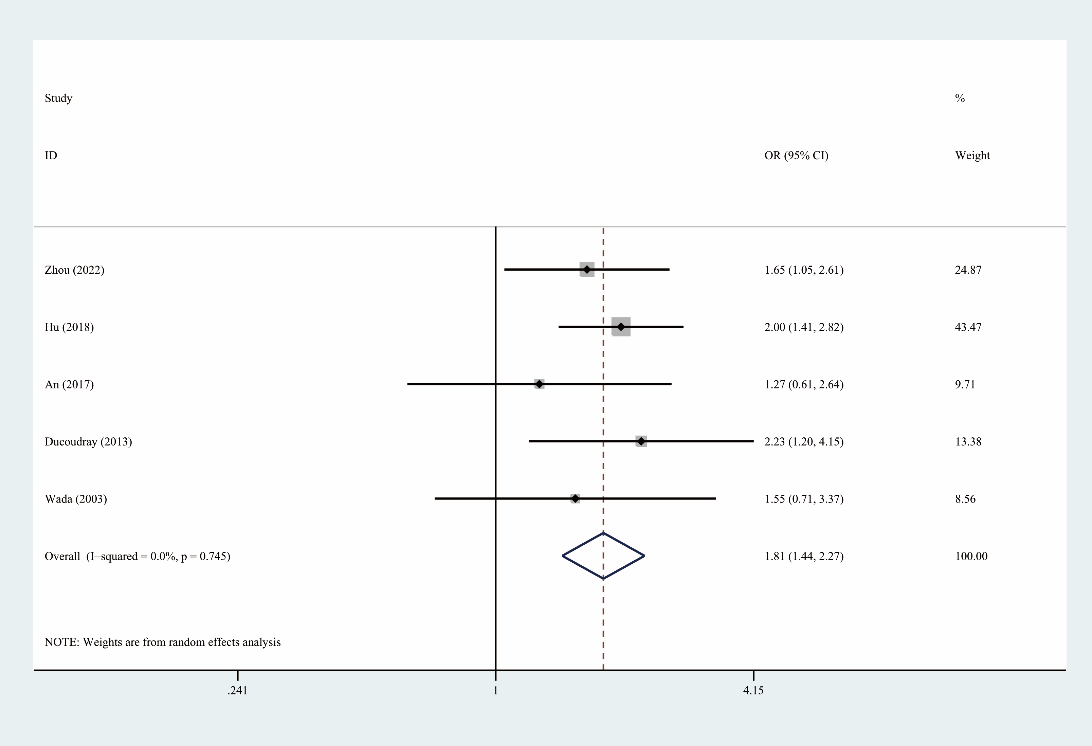


Figure S10. A random-effect model for the association of tumor located in upper pole with risk of OLLNM.


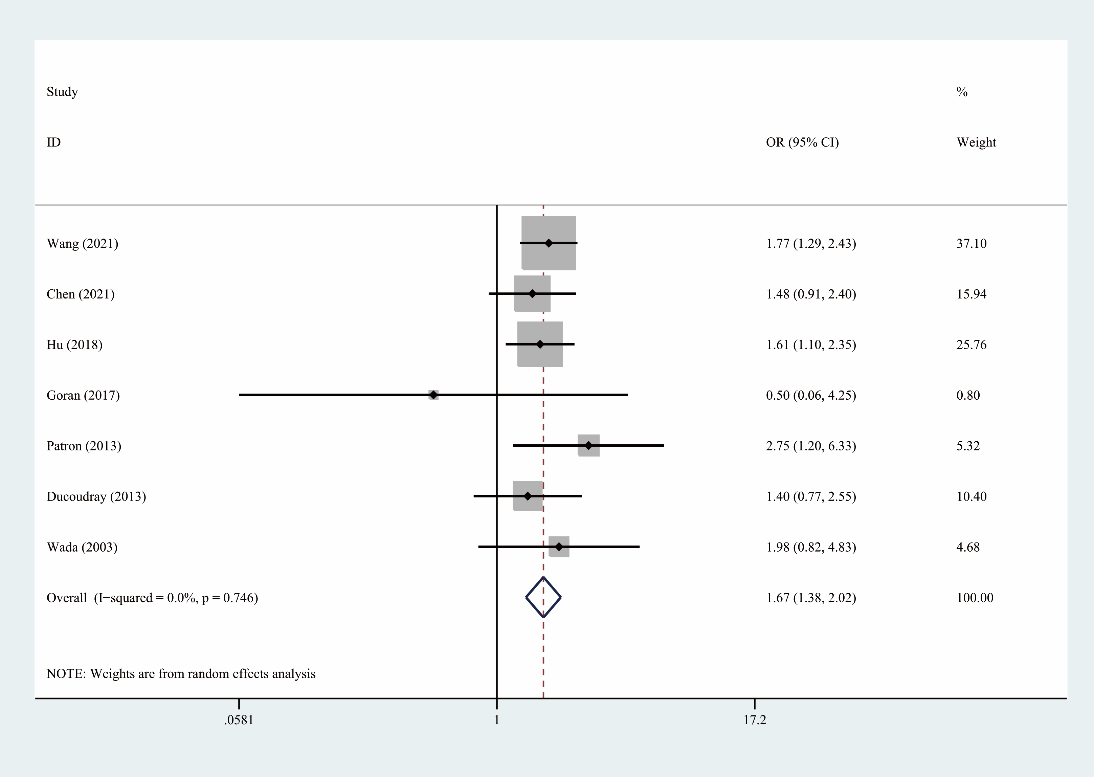


Figure S11. A random-effect model for the association of bilateral PTC with risk of OLLNM.


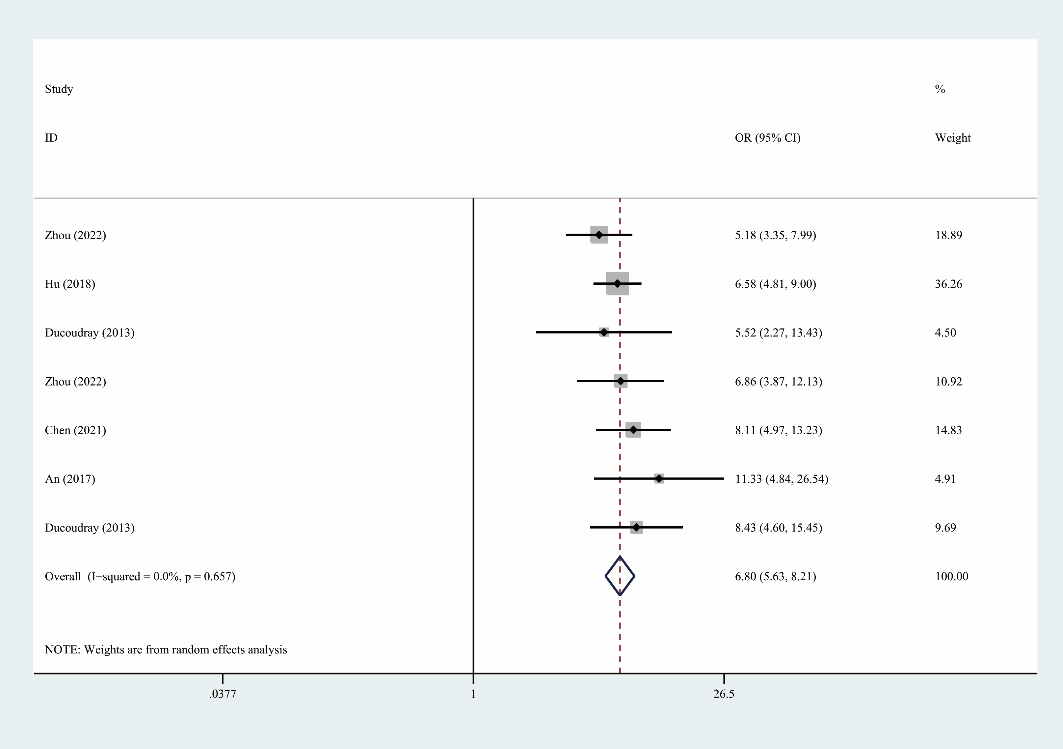


Figure S12. A random-effect model for the association of increased number of central lymph node metastasis (CLNM) with risk of OLLNM.


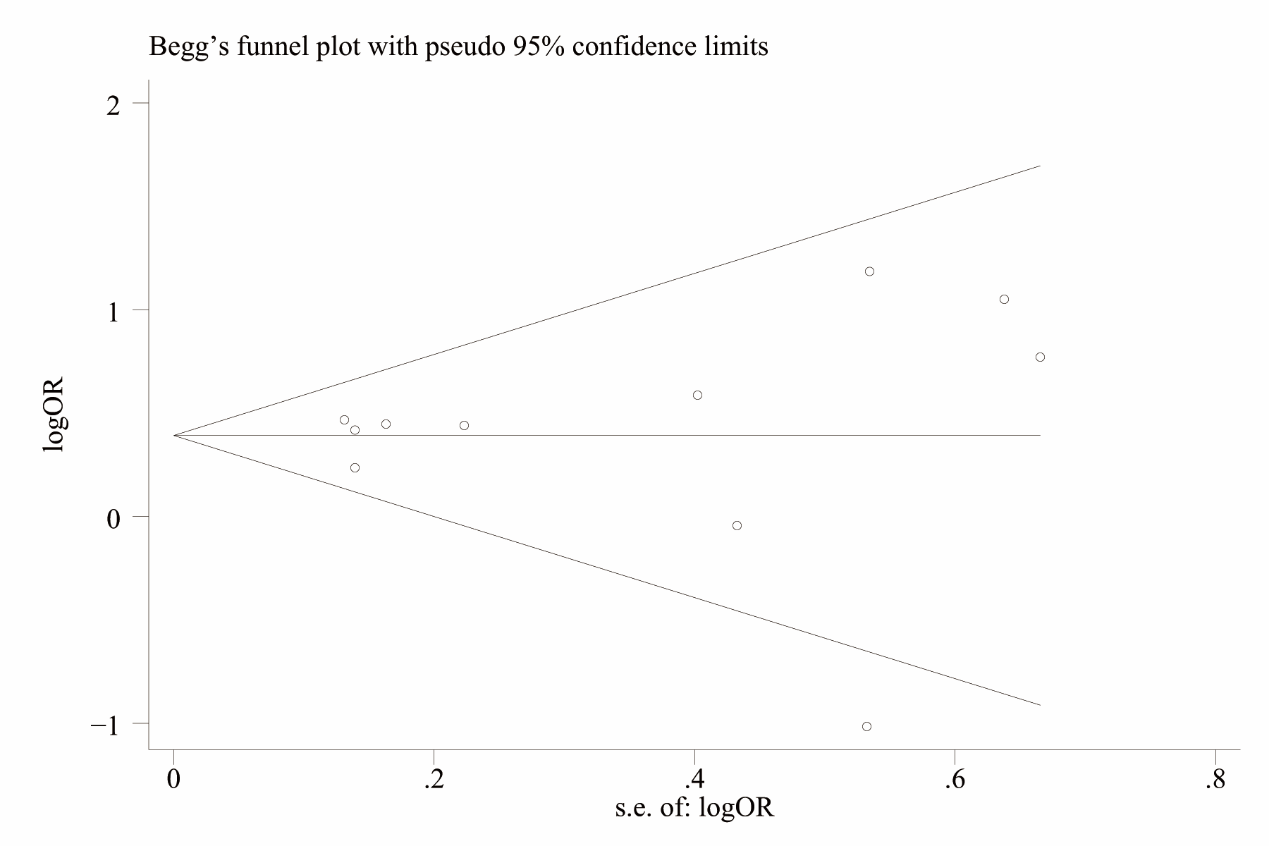


Figure S13. publication bias for the association of sex male with OLLNM (Begg's test: *P*=0.436; Egger’s test: *P*=0.951).


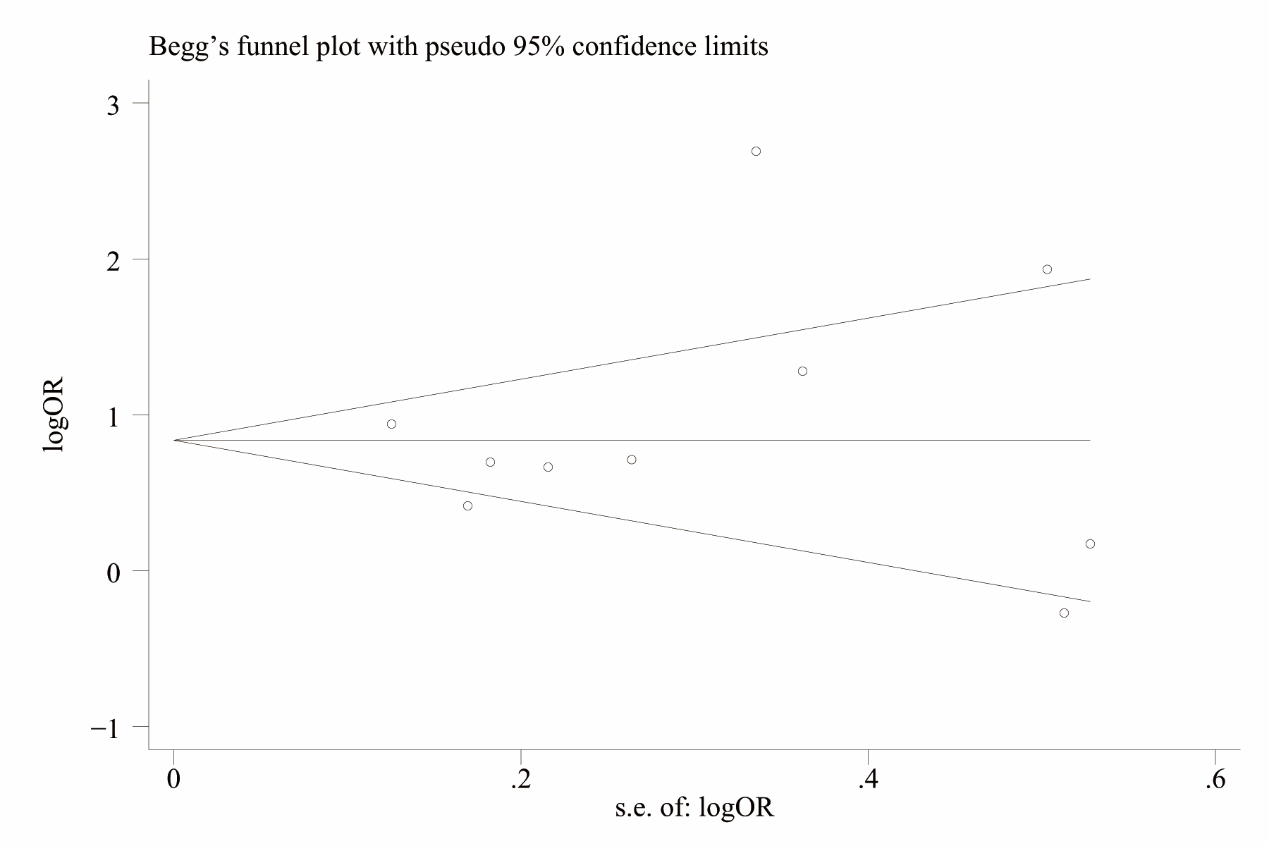


Figure S14. publication bias for the association of extrathyroidal extension (ETE) with OLLNM (Begg's test:*P*=0.858; Egger’s test: *P*=0.609).
